# Notch Signalling Inhibits CD4 Expression during Initiation and Differentiation of Human T Cell Lineage

**DOI:** 10.1371/journal.pone.0045342

**Published:** 2012-10-12

**Authors:** Stephen M. Carlin, Melissa L. M. Khoo, David D. Ma, John J. Moore

**Affiliations:** 1 Blood Stem Cells and Cancer Research, St Vincent's Centre for Applied Medical Research, Sydney, New South Wales, Australia; 2 Haematology Department, St Vincent's Hospital, Sydney, New South Wales, Australia; University of California, San Francisco, United States of America

## Abstract

The Delta/Notch signal transduction pathway is central to T cell differentiation from haemopoietic stem cells (HSCs). Although T cell development is well characterized using expression of cell surface markers, the detailed mechanisms driving differentiation have not been established. This issue becomes central with observations that adult HSCs exhibit poor differentiation towards the T cell lineage relative to neonatal or embryonic precursors. This study investigates the contribution of Notch signalling and stromal support cells to differentiation of adult and Cord Blood (CB) human HSCs, using the Notch signalling OP9Delta co-culture system. Co-cultured cells were assayed at weekly intervals during development for phenotype markers using flow cytometry. Cells were also assayed for mRNA expression at critical developmental stages. Expression of the central thymocyte marker CD4 was initiated independently of Notch signalling, while cells grown with Notch signalling had reduced expression of CD4 mRNA and protein. Interruption of Notch signalling in partially differentiated cells increased CD4 mRNA and protein expression, and promoted differentiation to CD4^+^ CD8^+^ T cells. We identified a set of genes related to T cell development that were initiated by Notch signalling, and also a set of genes subsequently altered by Notch signal interruption. These results demonstrate that while Notch signalling is essential for establishment of the T cell lineage, at later stages of differentiation, its removal late in differentiation promotes more efficient DP cell generation. Notch signalling adds to signals provided by stromal cells to allow HSCs to differentiate to T cells via initiation of transcription factors such as HES1, GATA3 and TCF7. We also identify gene expression profile differences that may account for low generation of T cells from adult HSCs.

## Introduction

HSC transplant is used to reconstitute the immune system after ablative therapy, but post-transplant the T cell lineage can be slow to recover [1]. While this is partly due to dependence on thymic activity, there is an intrinsic quality of adult cells which limits their T cell differentiation potential compared with CB cells in *in vitro* culture [2]. Understanding the mechanisms which drive T cell development is thus key to the development of strategies to improve transplant outcomes.

Notch signalling promotes a range of cell differentiation programs including neuronal and vascular fates [3], [4], [5], [6], [7], [8], and it is reported to be essential in three aspects of haematopoietic cell differentiation: maintenance of HSCs [9], [10], initiation of the T cell lineage [11], [12], [13], and maturation of CD4 and CD8 thymocytes [14], [15]. After multipotent CD34^+^ HSCs leave the bone marrow, generation of the T lineage is triggered by entry into the Notch signalling environment of the thymus. At an early differentiation stage, these lymphoid-primed multipotent precursors lose erythrocyte/megakaryocyte potential and initiate the lymphoid gene program, whilst maintaining myeloid potential [16]. Myeloid fates are subsequently inhibited by the Notch signalling environment [17]. In thymocytes, direct targets of Notch signalling such as HES1 are activated long before cell populations become committed to the T lineage, which demonstrates the lack of an early ‘lock down circuit’ [18] or clear binary switch to the T cell fate. Maturation of thymocytes then involves a complex interaction between transcription factors which control expression of Notch [19], [20], [21]. Thymocyte differentiation can be tracked by expression of cell surface markers such as CD7, CD1a and CD3 (in order of appearance) but the standard markers for human thymocytes are CD4 and CD8, which are first expressed singly in Immature Single Positive cells (ISPs), and then expressed together on Double Positive (DP) cells, which also co-express CD3 and proceed to selection by antigen testing.

Several methods are used to induce Notch signalling, and this may have led to discrepancies in the reported role of Notch signalling. Cells can be genetically modified to express constitutively active Intracellular Notch (ICN), co-cultured with stromal cells engineered to express Notch ligands, co-cultured with a mix of cells including Notch ligand-expressing cells (ie thymic organ culture [22] or keratinocyte/fibroblast mixes [23]), or cultured with Notch ligand in the absence of stromal support cells. Of these methods, co-culture with OP9 stromal cells expressing the Notch ligand Delta-like1 [24] is an established method with the endpoint of TCR^+^ CD3^+^ CD4^+^ CD8^+^ thymocytes from human HSCs.

Notch signalling, however, does not act alone in governing these cell fates. Culturing HSCs without stromal cells has demonstrated that the Notch signal alone does not direct cells to the T lineage, but rather expands HSC populations [25] to a cell type capable of rapid multi-lineage reconstitution in transplant recipients. The Wnt and Hedgehog pathways are also central to HSC maintenance and T cell lineage differentiation [26]. Other evidence suggests that Notch is dispensable for maintenance of adult HSCs, and that differentiation is governed by a range of Notch signalling intensities [27]. After induction of T lineage, Notch signalling is also reported to promote proliferation rather than differentiation [28]. Differentiation of T cells is also dependent on stromal cells, and cytokines such as Kit/SCF, IL-7 and Flt3 [29], [30], [31]. A recent review by Rothenberg [32] describes factors auxiliary to Notch which direct T lineage differentiation. TCF7 (TCF-1) is downstream of Notch, but forced expression drives T lineage differentiation in the absence of Notch, and once established, TCF7 positively autoregulates to maintain expression when Notch signals cease [33]. GATA-3 is also necessary for T lineage differentiation, and a T (and NK) cell specific enhancer region has recently been identified [34]. Bcl11b is necessary for commitment to T lineage and for later stages of development [21].

This study aimed to investigate the elements of the OP9Delta co-culture system with a view to improving the T cell yield of adult HSCs. We describe the differences between CB and adult HSCs in their T cell differentiation potential, and show that yield is increased by removing Notch signalling after lineage commitment.

## Results

### Effect of Notch signalling on T cell differentiation

**Figure 1:**
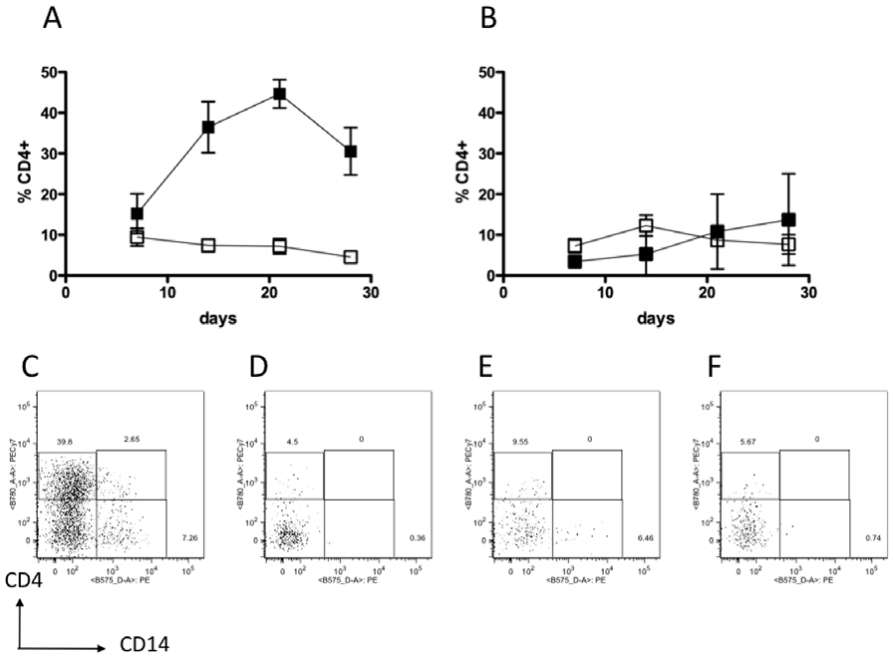
Expression of CD4 on cells co-cultured with or without Notch signaling. (A) CB and (B) Adult HSCs were maintained in OP9Delta^pos^ (□) or OP9Delta^neg^ (▪) co-cultures for 28 days with weekly analysis of CD4^+^ content by flow cytometry. Both CB and adult - derived HSCs expressed CD4 without Notch signaling. Data presented as mean ± SEM (n = 10 for OP9Delta^pos^ and n = 4 for OP9Delta^neg^). (C–F) Representative flow cytometry plots showing CD4 and CD14 expression on cells grown in co-culture for 28 days. (C) CB in OP9Delta^neg^, (D) CB in OP9Delta^pos^, (E) adult in OP9Delta^neg^, (F) adult in OP9Delta^pos^.

**Figure 2:**
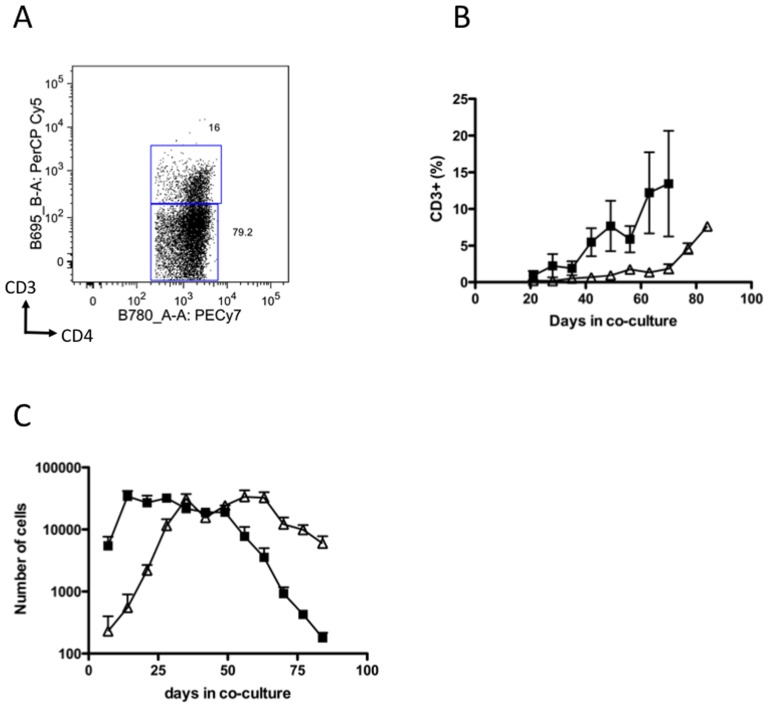
Growth of adult and CB cells on OP9Delta^pos^ co-culture. (A) Representative dot plot (Adult cells, 84 days, 7 days DAPT treatment) showing expression of CD3 and CD4 within the DP population. (B) Plot of CD3 generation by CB (▪) and adult (▵) cells in OP9Delta^pos^ co-culture. (C) Cell number in OP9Delta^pos^ CB (▪) and adult (▵) co-culture (mean ± SEM, n = 9).

Sorted HSCs (CD34^+^CD45^mid^Lin^−^) from adult or CB donors were grown in co-culture with Delta-like1-expressing OP9 cells (OP9Delta^pos^) or control OP9 cells (OP9Delta^neg^) and monitored by flow cytometry at weekly intervals. Without Notch signalling cells expressed CD19 and CD14, as expected from well-established reports [24], [35] indicating differentiation to B cell and monocyte lineages respectively (data not shown). A surprising result however was a high rate of cell surface expression of CD4 protein without Notch signalling (Figure 1 A & B). A higher proportion of cells expressed CD4 without Notch signalling in early CB cell co-cultures, and in adult cells the proportion of CD4-expressing cells was approximately equal with or without Notch signalling. In Notch-signalling OP9Delta^pos^ co-cultures, CB and adult cells differentiated to CD4 and CD8 ISP cells before becoming DP cells. A proportion of DP cells co-expressed CD3 indicating T lineage differentiation (Figure 2 A & B).

### Effect of Notch signal interruption on T cell differentiation

**Figure 3:**
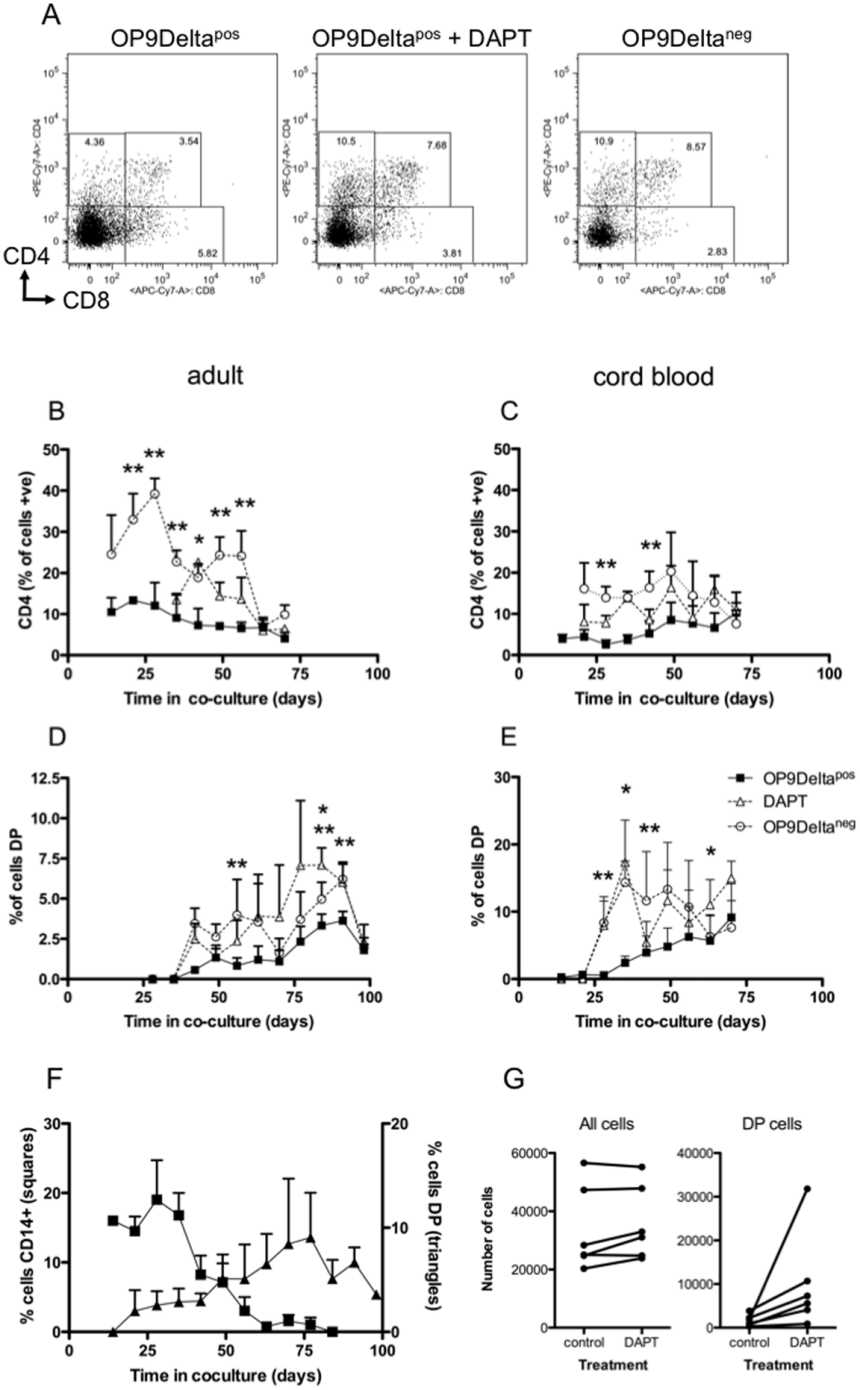
Notch signal interruption promotes CD4 and DP cell differentiation. CB and adult HSCs were grown in OP9Delta^pos^ co-culture, then Notch signalling was removed for 7 days by transfer to OP9Delta^neg^ co-culture or addition of DAPT. Cells were analysed by flow cytometry for CD4 and CD8 expression. (A) Representative dot plots for adult cells co-cultured for 70 days. (B–E) Adult (B, D) or CB (C, E) HSCs were grown in OP9Delta^pos^ co-culture (▪) and then either transferred to OP9Delta^neg^ co-culture (○) or incubated with DAPT (▵) for 7 days. Data points show the percentage of CD4^+^ ISP (B, C) or CD4^+^ CD8^+^ DP (D, E) cells, n≥3, mean+SEM, * *p*<0.05 for DAPT vs OP9Delta^pos^ (*t*-test), ** *p*<0.05 for OP9Delta^neg^ vs OP9Delta^pos^ (*t*-test). (F) CD14 (▪) expression plotted against DP expression (▴) for adult cells in OP9Delta^pos^ co-cultures after Notch signalling was removed for 7 days. (G) Cell numbers during DAPT treatment. Paired comparisons of six control and DAPT – treated cultures, showing all HSC-derived cells (left) and DP cells (right).

We also tested the effect of removal of Notch signalling on the differentiation of developing thymocytes (Figure 3). T lineage differentiation was induced by co-culturing HSCs with OP9Delta^pos^, then Notch signalling was interrupted at weekly timepoints by transferring cells to OP9Delta^neg^ co-culture or by addition of Notch inhibitor (DAPT) for 7 days. The two methods of Notch signal removal were employed to address the possibility of non-canonical Notch signalling through ligand binding without ICN generation, but both treatments resulted in the same effect. Representative dot plots are shown in Figure 3A. As shown in Figure 3B and C, interruption of Notch signalling for 7 days increased the percentage of cells expressing CD4 in both adult and CB co-cultures, but the effect was clearest in adult cells early in culture, (ie at 42 days, 7.5% CD4^+^ with Notch (OP9Delta^pos^), vs 20% CD4 with Notch removed (OP9Delta^neg^ and DAPT)).

Removal of Notch signalling also increased differentiation to the DP phenotype in cells derived from CB and adult precursors (Figure 3D and E). In adult cells the promotion of CD4 ISP cells gave way to direct progression to the more mature DP phenotype beyond 42 days in Notch co-culture (at 84 days, 3% DP with Notch, vs 7% DP with Notch removed). Early (days 28 to 42) DP differentiation of CB cells was significantly promoted by removal of Notch signalling, while later in culture (on a higher DP differentiation background) the effect of Notch removal was less. Increase in DP percentages could be due to either increased differentiation, or selective survival of DP cells while undifferentiated cells die due to removal of Notch signalling. Although cell number data is qualified in these assays due to possible losses of cells adherent to the OP9 cell layer in each passage, our results support increased differentiation rather than selective cell death (Figure 3G). At 42 days, mean total cell numbers for CB cells were; control 33 000, DAPT treated 36 000, while mean DP numbers were; control 1 700, DAPT treated 10 000 (n = 6). Similar findings were obtained with adult cells.

Adult cells also expressed the myeloid lineage marker CD14 in response to Notch interruption (Figure 3F) early in co-culture. However, beyond 42 days myeloid (CD14) differentiation in response to Notch removal was substantially lost, concomitant with the increase in DP differentiation, suggesting that this is a commitment point for the T lineage in adult cells. Expression of CD4 and CD14 were largely mutually exclusive (less than 5% of CD4^+^ cells were CD14^+^) indicating that withdrawal of Notch signalling did not induce a CD4^+^ myeloid lineage cell. Microscopically, stained cells grown on OP9Delta^pos^ had the characteristic morphology of small lymphocytes with thin cytoplasm and a dense round nucleus. CB cells by contrast did not generate CD14^+^ cells (<1%) after Notch signal interruption at any time point, (data not shown), demonstrating exclusion from non-T lineage at early exposure to Notch. CD19 (B cell lineage) was not expressed in cells which had been exposed to Notch signalling. CD3 and CD8 expression were not affected by Notch inhibition in either adult or CB co-cultured cells (data not shown). There was no change in cell number during the week of DAPT treatment compared to cells maintained on OP9Delta^pos^, indicating that the changes in phenotype were due to differentiation, rather than selective survival of differentiated cells. Cell numbers during co-culture on OP9Delta^pos^ are shown in Figure 2C.

### Gene expression in OP9 co-culture with and without Notch signalling

**Figure 4:**
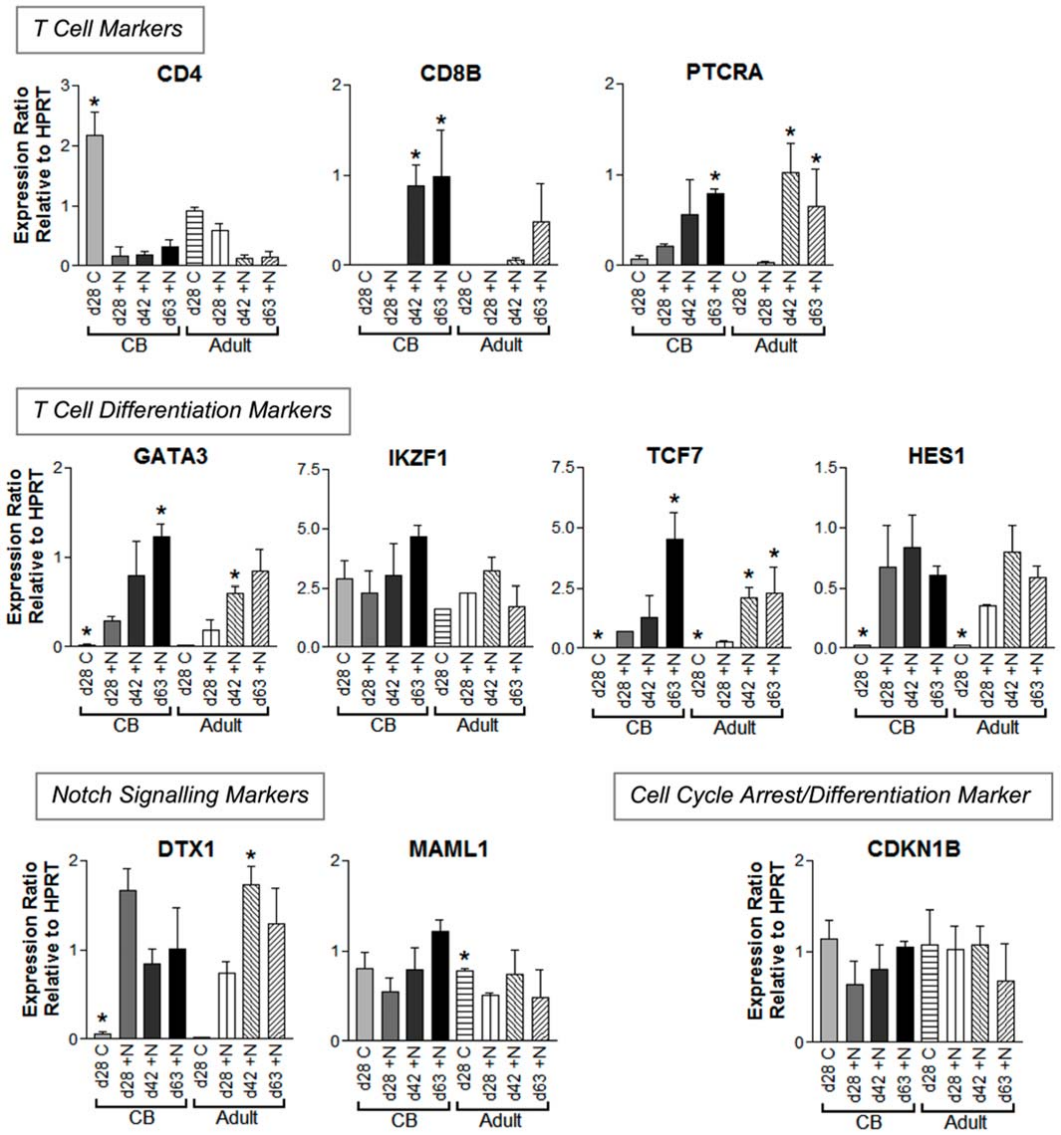
Gene expression differences in HSCs co-cultured on OP9Delta cells with or without Notch signalling. Real-time RT-PCR results from cord blood (CB) or adult HSCs co-cultured with either control OP9Delta^neg^ (C) cells, or OP9Delta^pos^ (+N) cells. Results are depicted as mean expression ratio +SEM relative to housekeeping gene *HPRT* (*n* = 3–4 for CB; *n* = 2–4 for adults). * *p*<0.05 compared with ‘d28 +N’ for CB and adult respectively, unpaired 2-tail *t*-test. d = day. PTCRA = pre-T cell antigen receptor alpha, GATA3 = GATA binding protein 3, IKZF1 = IKAROS family zinc finger 1, TCF7 = Transcription Factor 7 (T-cell specific, HMG-box), HES1 = Hairy and enhancer of split 1, DTX1 = Deltex homolog 1, MAML1 = Mastermind-like 1, CDKN1B = Cyclin-dependent kinase inhibitor 1B.

To resolve the effects of Notch signalling from the effects of stromal cell co-culture, we next compared gene expression in paired OP9/HSC co-cultures grown with or without Delta/Notch signalling (Figure 4). *CD4* was expressed in OP9Delta^neg^ co-cultures (CB and Adult d28 C), showing that initiation of this typically T cell gene is not governed by Notch signalling. In corresponding OP9Delta^pos^ co-culture (+N), *CD4* expression was lower in CB cells, and decreased at one-tail significance in adult. A set of T cell related genes (*TCF7*, *GATA3*, *HES1*, and Deltex homolog 1 (*DTX1*)) were clearly Notch-related as expression was detected in OP9Delta^pos^ co-culture but absent or only weakly expressed in OP9Delta^neg^ co-culture in both adult and CB cells. A subset of these (*TCF7* and *GATA3*) as well as *PTCRA* and *CD8B* also increased with time in OP9Delta^pos^ coculture. Expression levels of *GATA3* (at 63 days, n = 3) and *CD8B* (at 42 days, n = 3) were significantly higher in CB relative to adult cells (p<0.05, two tailed *t*-test). IKAROS family zinc finger 1 (*IKZF1*) and Mastermind-like 1 (*MAML1*) were expressed in developing HSCs regardless of Notch signalling, and whilst *IKZF1* expression was not initiated by Notch signalling, a trend of increasing expression with time in OP9Delta^pos^ co-culture was observed in CB co-cultures.

### Effect of Notch signal removal on T lineage mRNA expression

**Figure 5:**
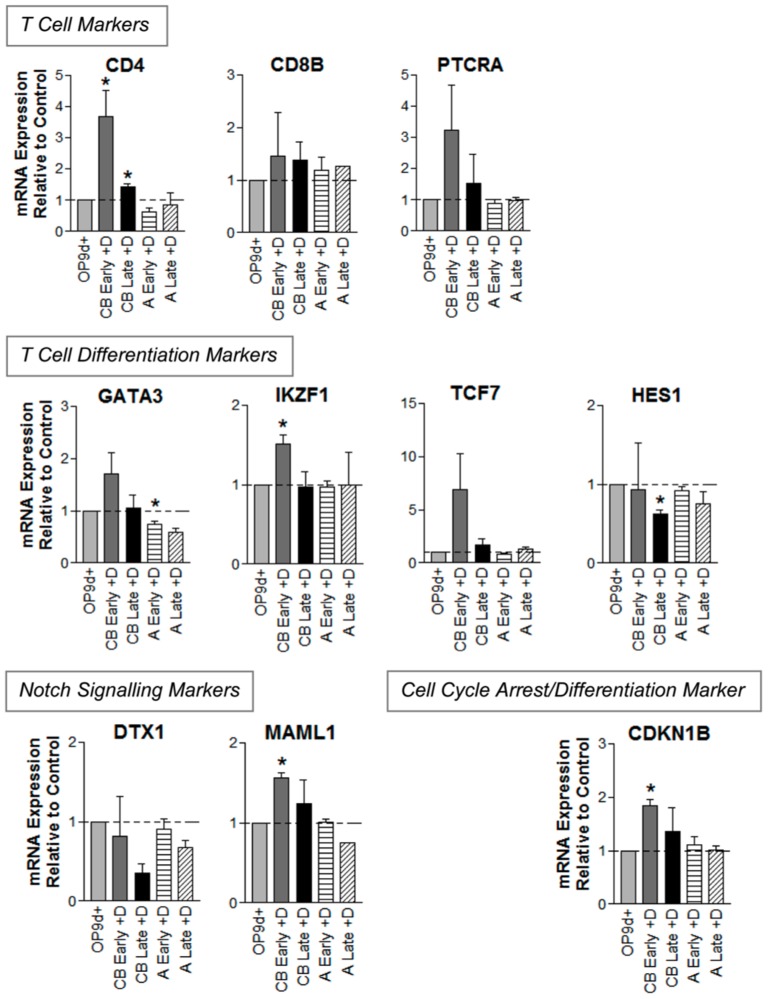
Effect of Notch signal removal (DAPT) on mRNA expression of CB and adult HSCs undergoing T lineage differentiation. Real-time RT-PCR results from cells grown in OP9Delta^pos^ co-culture with Notch inhibitor (DAPT) added for 7 days before harvesting. Timepoints: CB (cord blood) early = 42 days, CB late = 56 days, A (adult) early = 42 days, A late = 70 days. Data are presented as mean fold change in mRNA expression + SEM relative to untreated control cultures (OP9Delta^pos^), with baseline set at 1.0 (*n* = 3 for CB; *n* = 2–4 for adults). * *p*<0.05 compared with the control, paired 2-tail *t*-test.

To investigate gene expression changes caused by interruption of Notch signalling, we examined the expression of a suite of T lineage development-related genes during OP9Delta^pos^ co-culture at times chosen for maximal promotion of differentiation (42 days for CB (‘Early’) and 70 days for adult cells (‘Late’), Figure 5). For CB cells, interruption of Notch signalling for the final 7 days of culture significantly promoted *CD4* mRNA expression by greater than 3 fold in early co-cultures (42 days). *CD4* expression was also significantly increased with Notch inhibition in late co-cultures (56 days), although on a background of higher basal *CD4* expression (0.33×*HPRT* (d63), cf 0.18×*HPRT* (d42), *p*<0.05, Figure 4). *IKZF1*, a gene involved in T cell differentiation, was also significantly increased by Notch interruption in CB cells, together with Notch signaling marker *MAML1*, and cell cycle arrest/differentiation marker Cyclin-dependent kinase inhibitor 1B (*CDKN1B*; also known as *p27*, *Kip1*). Like *CD4*, basal expression of *IKZF1*, *MAML1*, and *TCF7* increased between 42 and 56 days. Notch interruption also decreased *HES1* and *DTX1* expression in CB cells at 56 days, whilst *CD8B* expression was not affected at any time point. Notch interruption always increased expression of *TCF7* and *GATA3* at the early timepoint in CB cells, although this was not statistically significant at 2 tailed *t*-test due to the wide spread of increase (2 fold to 13 fold for *TCF7*). This does however demonstrate that although they were only expressed in Notch signalling cells, continued expression of *TCF7* and *GATA3* was not reliant on Notch.

The effect of Notch interruption on gene expression in adult cells was markedly different. Although Notch inhibition caused a significant increase in CD4 protein expression (OP9Delta^neg^ co-cultures at 24–42 days in Figure 3B), this was not reflected in an increase in *CD4* mRNA expression at any time. Furthermore, T cell differentiation markers *IKZF1* and *TCF7* were not upregulated, and *GATA3* was significantly decreased. In addition, little change was observed in both *MAML1* and *CDKN1B*, unlike the significant increases observed with Notch interruption in CB cells.

## Discussion

Notch signalling is central to T cell differentiation, but here we have found it is associated with reduced gene and protein expression of the prime T cell protein CD4. CD4 mRNA and protein expression was initiated in OP9Delta^neg^ (non– Notch signalling) co-cultures, and initiated but expressed in lower amounts in OP9Delta^pos^ co-cultures. In cells that had begun T cell differentiation, interruption of Notch signalling promoted expression of CD4 protein early in co-culture, and promoted differentiation to the CD4^+^CD8^+^ DP phenotype in late co-culture. Notch signalling also, as expected, inhibited expression of markers of non-T cell lineages such as CD19 and CD14.

By comparing cells grown in OP9Delta^pos^ and OP9Delta^neg^ co-cultures we identified four genes (*TCF7*, *GATA3*, *HES1*, and *DTX1*) that were initiated by Notch signalling. Only *HES1* and *DTX1* remained in direct positive Notch control (down-regulated by Notch inhibitor DAPT), and expression of *CD8B*, *PTCRA*, *TCF7* and *GATA3* appeared to be part of a general change of transcriptome involved in T cell differentiation. The suite of genes up-regulated in more differentiated CB cells (late OP9Delta^pos^ co-culture) compared to early CB cells, namely *MAML1*, *TCF7*, *IKZF1*, *GATA3* and *CD4*, may be a signature of differentiated T cells. With interruption of Notch signalling, *CD4*, *IKZF1*, *MAML1* and *CDKN1B* showed significantly increased expression in CB co-cultures, while *HES1* was significantly decreased, and *TCF7* and *GATA3* showed an increasing trend. A markedly different response was observed in adult co-cultures, suggesting a molecular basis for the observed differences in T cell generation between CB and adult HSCs.

The initial expression of *CD4* and *CD8* genes are reported to be controlled by BAF complexes and chromatin remodelling [36]. Subsequent fine control of *CD4* expression is managed by a promoter element and a silencer activated by RUNX1 (runt-related transcription factor 1) [37], [38]. Our results show that initial *CD4* expression was activated by stromal cell contact (in both OP9Delta^neg^ and OP9Delta^pos^ co-cultures). In the absence of Notch signalling (OP9Delta^neg^) CB cells showed increased CD4 protein expression, while growth in the presence of Notch signalling maintained CD4 protein expression at low levels. These results are consistent with a role for Notch in driving the CD4 silencer element. The *HES1* transcription factor is reported to control *CD4* expression by binding to the *CD4* silencer [39], although this finding has been disputed [40]. We found that *HES1* was weakly expressed in both adult and CB OP9Delta^neg^ co-culture, when *CD4* was upregulated; while in the presence of Notch signalling, *HES1* expression was initiated and *CD4* expression was downregulated. Furthermore, after DAPT inhibition of Notch signalling, *HES1* expression was decreased and *CD4* expression was significantly increased in CB co-cultures. Taken together, these findings suggest that Notch signalling controls *CD4* expression via regulation of *HES1* expression.

At later stages of thymocyte differentiation, *CD4* expression is reported to be promoted by TCF7 and GATA3 through binding to the proximal enhancer of the *CD4* gene [41] and de-repressing CD4 expression [42] respectively. The data in this study support a positive relationship between GATA3/TCF7 expression and the CD4 proximal enhancer in the mediation of CD4 expression. Although *GATA3* and *TCF7* expression were initiated in a Notch signalling environment, in CB cells these genes also increased in response to Notch interruption. This suggests that in T cell differentiation, during the late stages of the DN-DP transition, factors other than Notch signalling are involved in promoting *GATA3* and *TCF7* expression. Expression of *GATA3* was significantly lower in adult cells compared to CB, and this may partly explain the lower proportion of CD4^+^ adult cells generated. Reports have also suggested that *IKZF1* may have a role in antagonizing Notch signalling [43], and this is reflected in our findings of increasing *IKZF1* expression during OP9Delta^pos^ co-culture

The mechanism by which Notch signalling promotes T cell differentiation has not yet been defined, although gene expression array data for Notch-induced genes have been published [44], [45]. Notch signalling has been reported to be downregulated during T cell maturation [46] and to be not required for development post-b-selection [28] [47]. Pre-TCR signalling has been shown to inactivate Notch transcription at the transition from DN to DP [48], and down-regulation of Notch signalling post-b-selection may protect against undesirable proliferation. Reduction of high Notch signalling early in T lineage commitment has also been found to promote the major alpha-beta TCR lineage over the gamma-delta TCR [49]. These results support a role for Notch in maintaining low levels of both CD4 mRNA and protein expression during early T cell differentiation. Stromal cell contact was also critical for activation of genes central to the T cell lineage, in particular CD4. Our results are consistent with adult HSC-derived cells having low activation of *GATA3* and *TCF7*, which have a critical role in T lineage specification and differentiation [33], in comparison with CB-derived cells, and a consequent reduced ability to up-regulate CD4 expression for completion of T cell maturation. The lack of CD4 mRNA response to Notch inhibition in adult cells cannot be explained from our results. As CD4 protein responded to Notch inhibition, and CD4 mRNA expression otherwise correlated with protein expression in adult cells, we speculate that the adult cells responded to DAPT with a transient increase in mRNA expression that was not detected by analysis at 7 days, while CD4 protein expression was maintained on cells and was detected by flow cytometry. CB cells by contrast continued to respond to DAPT throughout the incubation.

In conclusion, this study finds that inhibition of Notch can allow for efficient differentiation of thymocytes to the DP stage. In addition, this study reaffirms the previously reported intrinsic molecular and phenotypic differences between cord and adult cells in their generation of the T cell lineage [2]. It is possible that the distinct molecular responses to Notch of cord cells provides them with an increased T cell regenerative potential compared to adults but further studies will be required to fully characterise this difference.

## Materials and Methods

### Precursor cells

The study was approved by the Human Research and Ethics committee, St Vincents Hospital (Study No. H06/148). GCSF-mobilized (5 days) peripheral blood from normal adult donors was cryostored in 10% DMSO after informed written consent. Cells no longer needed for clinical use were thawed, then washed in PBS 1% BSA. Lineage cells were depleted with a magnetic bead separation kit (Miltenyi). Cells were stained for CD45, CD34 and Lineage (CD3, CD14, CD16, CD19, CD20, and CD56), (all antibodies from BD), then FACS sorted (FACS Diva, Becton Dickinson). Primary gating was for SSC^lo^, CD45^mid^, then for Lin^−^, CD34^+^. Sorted cells were seeded at 3 000 per well (24 well plate) into OP9 co-cultures, which were maintained for up to 100 days.

CB samples were fractionated to peripheral blood mononuclear cells (PBMC) using density centrifugation over Ficoll (GE Healthcare), and cryostored in 10% DMSO. Thawed cells were sorted as described for adult blood.

### Co-culture

OP9 cells [50] stably expressing Delta ligand (OP9Delta^pos^) and their control cells (OP9Delta^neg^) were obtained as a kind gift from Professor Zuniga-Pflucker (Sunnybrook Research Institute, Toronto, Canada) via Dr Gerard Hoyne (Australian National University, Canberra). Cells were maintained by the standard published method [24], grown in aMEM containing 20% FBS, and passaged 3× weekly by trypsinization. For co-culture, OP9 cells were seeded into 24 well plates at 75% confluence, and progenitor cells were added the next day in growth medium containing Flt3, IL-7 and SCF (all 5 ng/mL, from R&D Systems). Co-cultured precursors were passaged onto fresh OP9 cells at weekly intervals by vigorous pipetting and passing though a 70 mm strainer. Aliquots of the resuspended cells were taken for FACS analysis. For removal of Notch signalling, cells were transferred to OP9 Delta^neg^ wells, or to OP9Delta^pos^ wells with DAPT g-secretase inhibitor (Sigma) added at 5 mM, and grown for a further 7 days.

### Flow Cytometry

Cells were stained using the following antibodies; CD3 PerCP Cy5.5, CD4 PE Cy7, CD7 PE, CD8 APC Cy7, CD14 APC, CD19 PE, CD34 PE Cy7, CD45 APC Cy7, all from BD. Cells were incubated with antibodies for 10 min, then washed with PBS containing 0.2% NaN_3_ and 0.2% BSA, and resuspended in PBS containing 0.5% paraformaldehyde. Cells were analysed on an LSRII flow cytometer (BD). A primary gate of SSC^low^ FSC^midrange^ separated out developing haematopoietic cells, and surface marker expression was analyzed as a percentage of these.

### Statistics

Timecourse data were compared for significance using non-parametric one way repeated measures ANOVA (Friedman test) and Dunn's multiple comparison post hoc test (for 3 treatment variables), Wilcoxon matched pair test, or Mann Whitney U test, as stated in the figures. Significance at p<0.05 was assumed for analyses. Data analyses were performed using SPSS version 15 statistical software. Graphics were produced using Graphpad Prism version 3.0.

Gene expression data were expressed as mean fold change in mRNA expression+SEM relative to untreated control cultures, or mean expression ratio+SEM relative to reference gene *HPRT*. The *t*-test was performed to determine significant differences (*p*<0.05) using Microsoft Excel. Linear correlations were tested for non-zero gradient using Pearson correlation coefficient.

### RNA Extraction and Real-Time PCR

Total RNA was extracted using TRIzol reagent (Invitrogen, CA, USA) and RNeasy micro kit with DNase I treatment (Qiagen, Basel, Switzerland). Total RNA (Adult: 90–230 ng; CB: 135–400 ng) was reverse transcribed with Superscript III (Invitrogen) using 25 ng random hexamers and 2.5 µM oligo(dT)_20_ primers, according to manufacturer's recommendations, and with RNase H treatment. Real-time RT-PCR was performed using Platinum SYBR Green qPCR SuperMix-UDG (Invitrogen) on a Rotor-Gene RG3000 machine (Corbett Research, NSW, Australia). The thermal profile for all reactions was: 2 min at 50°C, 2 min at 95°C, 40 cycles of 30 seconds (s) at 95°C, 30 s at 60°C and 30 s at 73°C. The ratio of the target gene expression in experimental/control (‘fold change in target gene’/‘fold change in reference gene’) was determined using the ΔΔCt method [51]. The reference gene used was hypoxanthine phosphoribosyltransferase 1 (*HPRT1*). To exclude genomic DNA contamination, RNA was treated with RNase-free DNase I (Qiagen) and primers were intron-spanning. Primers are listed in Table 1.

**Table 1:** PCR primers.

Gene	Sequence (5′-3′)	
	Sense	Antisense
***CD4***	actcaagaagaagcctttggga	cttggacacctacattgcactg
***CD8B***	agaggtggaacaggagaagatagc	agggtggacttcttggtggg
***PTCRA***	catctctccctgccttctgagg	gcaggtcaaacagcagcagc
***GATA3***	agccactcctacatggacgc	aaggggctgagattccaggg
***IKZF1***	tgccaagactccacggacacc	ttcatctgctccccgctg
***TCF7***	agccagaagcaagttcacagg	gcctccttctctgccttggac
***MAML1***	tgacaagccttctggagccg	ttgcgatatggagccgacagtc
***HES1***	ccgatggccagtttgctttc	ctggaaggtgacactgcgttg
***DTX1***	tggtcacagcatcaggctac	tggtctgggtatcagggaagc
***CDKN1B***	tccggctaactctgaggacac	aggtcgcttccttattcctgc
***HPRT1***	gaccagtcaacaggggacat	cctgaccaaggaaagcaaag
